# Glucocorticoid dysfunction in children with severe malaria

**DOI:** 10.3389/fimmu.2023.1187196

**Published:** 2023-07-10

**Authors:** Leen Vandermosten, Fran Prenen, Balotin Fogang, Pauline Dagneau de Richecour, Sofie Knoops, Christiane Josiane Donkeu, Cathy Doric Piemba Nguefack, Jean-Voisin Taguebue, Paul Koki Ndombo, Bart Ghesquière, Lawrence Ayong, Philippe E. Van den Steen

**Affiliations:** ^1^ Laboratory of Immunoparasitology, Department of Microbiology, Immunology and Transplantation, Rega Institute for Medical Research, KU Leuven, Leuven, Belgium; ^2^ Malaria Research Unit, Centre Pasteur du Cameroun, Yaoundé, Cameroon; ^3^ Mother and Child Center, Chantal Biya Foundation, Yaoundé, Cameroon; ^4^ Metabolomics Expertise Center, Center for Cancer Biology, VIB Center for Cancer Biology, Leuven, Belgium; ^5^ Metabolomics Expertise Center, Department of Oncology, KU Leuven, Leuven, Belgium

**Keywords:** malaria, glucocorticoids, plasmodium, metabolomics, cortisol

## Abstract

**Introduction:**

Malaria remains a widespread health problem with a huge burden. Severe or complicated malaria is highly lethal and encompasses a variety of pathological processes, including immune activation, inflammation, and dysmetabolism. Previously, we showed that adrenal hormones, in particular glucocorticoids (GCs), play critical roles to maintain disease tolerance during *Plasmodium* infection in mice. Here, GC responses were studied in Cameroon in children with uncomplicated malaria (UM), severe malaria (SM) and asymptomatic controls (AC).

**Methods:**

To determine the sensitivity of leukocytes to GC signaling on a transcriptional level, we measured the *ex vivo* induction of glucocorticoid induced leucine zipper (GILZ) and FK506-binding protein 5 (FKBP5) by GCs in human and murine leukocytes. Targeted tracer metabolomics on peripheral blood mononuclear cells (PBMCs) was performed to detect metabolic changes induced by GCs.

**Results:**

Total cortisol levels increased in patients with clinical malaria compared to AC and were higher in the SM versus UM group, while cortisol binding globulin levels were unchanged and adrenocorticotropic hormone (ACTH) levels were heterogeneous. Induction of both GILZ and FKBP5 by GCs was significantly reduced in patients with clinical malaria compared to AC and in malaria-infected mice compared to uninfected controls. Increased activity in the pentose phosphate pathway was found in the patients, but this was not affected by *ex vivo* stimulation with physiological levels of hydrocortisone. Interestingly, hydrocortisone induced increased levels of cAMP in AC, but not in clinical malaria patients.

**Discussion:**

Altogether, this study shows that patients with SM have increased cortisol levels, but also a decreased sensitivity to GCs, which may clearly contribute to the severity of disease.

## 1 Introduction

Malaria is a parasitic disease that poses a major health burden to the world with an estimated 247 million cases and 619,000 deaths in 2021 (1). Infection with *Plasmodium* parasites has diverse outcomes ranging from asymptomatic in semi-immune individuals to uncomplicated and severe malaria. Complications of malaria are the major cause of death and include cerebral malaria, severe malarial anemia, placental malaria and malaria-associated acute respiratory distress syndrome (MA-ARDS). The pathogenesis of malaria complications is complex and multifactorial, including processes such as parasite sequestration and inflammation (2). Moreover, metabolic disturbances including hypoglycemia and hyperlactatemia are common in malaria and contribute significantly to the severity of disease, but remain poorly understood (3).

Endogenous glucocorticoids (GCs) were found to be increased in patients with malaria as a result of stimulation of the hypothalamic-pituitary-adrenal (HPA) axis (4–6). GCs have potent anti-inflammatory and metabolic properties, which might help the host to tolerate disease (7). Due to their lipophilic properties, GCs cross plasma membranes and bind to the glucocorticoid receptor (GR). The GR is expressed in most cell types of the body, and when unliganded it resides in the cytoplasm, in complex with several chaperones including FK506-binding protein 5 (FKBP5). When the GC ligand binds, the GR dissociates from its chaperones and translocates to the nucleus, where it affects the transcription of a large number of genes, up to 10% of the genome (8, 9). This results in upregulation of the transcription of many molecules, including anti-inflammatory factors such as the glucocorticoid-induced leucine zipper (GILZ, encoded by *Tsc22d3*) and FKBP5, which inhibits the translocation of the GR and thus forms a negative feedback loop. Furthermore, liganded GR downregulates a variety of inflammatory factors and regulates several metabolic processes (9, 10). Interestingly, GCs also have less well characterized rapid transcription-independent effects, the so-called non-genomic effects. The mechanisms of these non-genomic effects may involve an elusive membrane-bound receptor at the cell-surface, and may influence several signal transduction pathways, including by enhancing the levels of cAMP (11).

For many years, synthetic GCs have been used in the clinic, in particular for their broad anti-inflammatory properties, and are amongst the most-prescribed drugs. However, GCs are not always able to downregulate inflammation, as GC resistance often impedes their activities, as is well-documented in e.g. chronic obstructive pulmonary disease and steroid-resistant asthma (12, 13). Clinical trials to evaluate the therapeutic efficiency of dexamethasone in cerebral malaria were performed more than 30 years ago, with no success (14, 15). However, the reasons for this failure and the roles of endogenous GCs in malaria remain poorly understood.

Importantly, one older study reported a hyporesponsive HPA axis in a limited number of patients with severe malaria (4). Recently, our group demonstrated that adrenal hormones confer disease tolerance in malaria, since they protect against lethality without affecting pathogen load (16). This protective effect was observed in four different mouse models of malaria with a disease severity ranging from asymptomatic or mild to lethal hyperparasitemia or lethal MA-ARDS. Infection of adrenalectomized mice with *Plasmodium* resulted in severe hypoglycemia and excessive inflammation in the circulation and brain, and this phenotype could be rescued by treatment with dexamethasone, a synthetic GC. Metabolic alterations and a hyperinflammatory state are often seen as independent entities in malaria. However, GCs seem to be central in mice to regulate both metabolism and immune responses.

In the current study, we investigated the GC response in children with severe malaria (SM) *versus* uncomplicated malaria (UM) and asymptomatic controls (AC). Plasma levels of cortisol were increased in the patients, while cortisol binding globulin (CBG) and adrenocorticotropic hormone (ACTH) were not different or highly variable. By analyzing the *ex vivo* induction of GILZ and FKBP5 mRNA by physiological levels of GCs, we found a reduced sensitivity of the peripheral blood mononuclear cells (PBMCs) from UM and SM patients compared to AC. Furthermore, metabolomics analysis indicated that cAMP and AMP levels were increased by GC treatment in PBMCs from AC, but not from UM or SM patients. Altogether, this study shows that patients with SM have increased cortisol levels but reduced transcriptional and non-transcriptional sensitivity to GCs.

## 2 Materials and methods

### 2.1 Ethics statement

All procedures were approved by the UZ Leuven Ethical Committee (Protocol number S62395). In Cameroon, ethical and administrative approvals were obtained from the National Ethics Committee for Research in Human Health (CNERSH) in Yaoundé (Protocol number 2019/03/1150/CE/CNERSH/SP) and the Ministry of Public Health. Prior to enrollment into the study, a written informed consent was obtained from the legal guardian or parent of each child according to the Declaration of Helsinki.

### 2.2 Study population

Samples for this cross-sectional study were collected from children aged 6 months to 17 years between October 2019 and July 2021, during two seasons of high transmission in a region with continuous *Plasmodium* transmission. Malaria cases were recruited from children who presented in the Chantal Biya Foundation Mother and Child pediatric referral hospital in Yaoundé. Asymptomatic children were recruited from the community and from children attending the Centre Pasteur du Cameroun for routine check-ups and vaccinations. They tested negative on the *Plasmodium falciparum* histidine rich protein II (PfHRPII) and pan-*Plasmodium* species-based rapid diagnostic tests (RDT) (SD Bioline, S. Korea).

Exclusion criteria were the administration of antimalarial drugs, corticosteroid drugs or vaccines during the last week before sampling, food intake within the last 2 hours, acute meningitis or other non-malaria severe illness within the last 48 hours, developmental delay, chronic illnesses or pregnancy. Furthermore, AC were excluded if they had symptoms of illness the past 4 days. All samples were collected between 7 am and 3 pm.

Inclusion criteria for malaria cases were the presence of *Plasmodium falciparum* parasites, initially tested by RDT. After inclusion, thick blood smears were examined to confirm the presence of *Plasmodium ssp* and determine the parasitemia. Uncomplicated malaria was defined as the presence of fever or history of fever in the last 48 hours in absence of severe malaria symptoms. Severe malaria is defined as one or more of the following symptoms: impaired consciousness (Blantyre score<3 or Glasgow score<10), prostration, 2 or more convulsion episodes within the last 24 hours, clinical manifestation of respiratory distress, severe anemia (hemoglobin<5 g/dl) and hyperparasitemia (>250,000 asexual parasites/µl). All children received standard clinical care after blood withdrawal.

### 2.3 Clinical parameters and sample processing

Weight, height, blood pressure and temperature were recorded from each included individual. Z-scores for malnutrition were defined based on WHO standards: weight-for-height for children younger than 5 years and BMI-for-age for children older than 5 years (17). Blood pressure Z-scores were calculated from percentiles obtained from 2004 blood pressure for age and height charts of the National Heart, Lung and Blood institute (NHLBI) (18). Venous blood was collected in EDTA-coated vacutainer tubes (BD, Franklin Lakes, NJ, USA). Lactate and glycemia levels were immediately measured on the blood that remained in the catheter, with a Lactate Plus meter (Cardioworld, Gärtringen, Germany) and an Accu-Chek Guide meter (Roche Diagnostics, Basel, Switzerland).

Whole blood was diluted in Türk’s solution (Merck, Darmstadt, Germany) and the white blood cell (WBC) concentration was determined using a Bürker chamber. Giemsa-stained thick blood smears were prepared and the number of parasites were microscopically counted against 1,000 WBCs. The parasite density (parasites/µl) was calculated from both counts instead of assuming an average count of WBC per µl of blood. Blood hemoglobin levels were assessed using a Mission Hb haemoglobinometer (ACON Laboratories, USA).

Plasma was isolated within 30 min after sample collection by 10 min centrifugation at 2,000 g and aliquots were stored at -80°C until further use. The pellet was diluted twofold in sterile Dulbecco′s Phosphate Buffered Saline (DPBS; Lonza, Verviers, Belgium), without Ca^2+^ and Mg^2+^ and with 2% Fetal Calf Serum (FCS; Gibco) and left gently shaking for maximally 6h. For the density-gradient purification of PBMCs, the diluted pellet was slowly pipetted on top of Pancoll (1.077 g/ml) (PAN-Biotech, Aidenbach, Germany) in a 1:3 volume ratio. After centrifugation for 30 min at 600 g (20°C) without brake, the layer with PBMCs above de Pancoll layer was transferred to a new tube, washed twice with DPBS and counted in Türk’s solution.

### 2.4 Plasma analyses

Plasma insulin and glucagon levels were assayed using ELISA (Mercodia, Uppsala, Sweden) according to the manufacturer’s protocols. Total levels of cortisol (Beckman Coulter, CA, USA), cortisol-binding globulin (CBG) (DIAsource, Louvain-la Neuve, Belgium) and adrenocorticotropic hormone (ACTH) (BRAHMS Diagnostics, Hennigsdorf, Germany) were measured using RIA following the manufacturer’s instructions. Albumin was determined with the colorimetric Bromocresol Purple assay (Sigma-Aldrich).

Based on total plasma cortisol, CBG, and albumin concentrations, free cortisol levels were calculated with the Coolens method, which was adapted for individual albumin levels as described by Boonen et al. (19, 20).


$$Free\;cortisol\;(\mu M)=\sqrt{{\left(0.0167\;+\;(G-T)\;\frac{1}{2(1+N\prime \prime )}\right)}^{2}\;+\;T\;*\frac{1}{(1+N\prime \prime )\;*\;K}}\;-0.0167\;+\;(G-T)\;\frac{1}{2(1+N\prime \prime )}$$

where G = plasma CBG concentration (µM), T = plasma total cortisol concentration (µM), K = affinity of CBG for cortisol = 30 µM^−1^, and N″ = 1.74/43 × individual albumin concentration (g/l).

### 2.5 Incubation of PBMCs with hydrocortisone

PBMCs were suspended at a concentration of 1x10^6^ cells per ml in RPMI-1640 medium (Caisson Labs, Smithfield, UT, USA) with 2 g/L sodium bicarbonate, 2 mM L-glutamine (Gibco) and 2% FCS (Gibco). The cells were plated in duplicate in 96-well plates with 250,000 cells per well and one well was treated with 150 nM hydrocortisone (Sigma-Aldrich). After 2h in a 5% CO2 incubator set at 37°C, cells were transferred to an Eppendorf. The well was washed with 0.9% NaCl and the adhering cells were lysed in 350 µl RLT with β-mercaptoethanol. Suspension cells were spun down, the cell pellet was lysed with the RLT from the plate and lysates were stored at -80°C until RNA extraction.

### 2.6 Murine splenocyte stimulation with corticosterone

C57BL/6J mice were bred in the animal house of the Rega Institute for Medical Research, KU Leuven. The mice were infected by intraperitoneal (i.p.) injection of 10^4^ red blood cells infected with the Edinburgh strain of *Plasmodium berghei* NK65 (PbNK65) (21). Non-infected controls from the same sex and age were included in each experiment. All experiments were performed according to the regulations of the European Union (directive 2010/63/EU) and the Belgian Royal Decree of 29 May 2013, and were approved by the Animal Ethics Committee of the KU Leuven (License LA1210186, project P123/2022, Belgium). Mice were euthanized by i.p. injection of 100 µl of Dolethal (Veítoquinol, Aartselaar, Belgium; 200 mg/ml). Spleens were removed and collected in ice cold PBS + 2% FCS and mashed through a 70 µm nylon cell strainer to obtain single cells. RBC lysis was by incubation in a 0.83% NH_4_Cl (Acros Organics, Geel, Belgium), 10 mM Tris (Sigma-Aldrich, Bornem, Belgium), pH 7.2 solution for 3 min at 37°C. The cells were then washed three times in PBS + 2% FCS and counted in a Bürker chamber with trypan blue exclusion of dead cells. Cells were resuspended at 2x10^6^ cells per ml in RPMI-1640 medium (Biowest, Nuaillé, France) with 10% FCS and plated in 24-well plates with 3x10^6^ cells per well. Per mouse sample, one well was left untreated and the other well was treated with 400 nM corticosterone (Sigma-Aldrich). After 2h in a 5% CO2 incubator set at 37°C, suspension cells were transferred to an Eppendorf and the adhering cells were lysed in 350 µl RLT from the RNeasy kit (Qiagen, Hilden, Germany) with β-mercaptoethanol. Suspension cells were spun down, the cell pellet was lysed with the RLT from the plate and lysates were stored at -80°C until RNA extraction.

### 2.7 RNA extraction and quantitative reverse transcription-polymerase chain reaction

RNA from PBMCs and splenocytes was extracted, respectively, using the RNeasy Micro Kit (Qiagen) and the RNeasy Mini Kit (Qiagen) according to the manufacturer’s protocol. RNA concentration was evaluated and RNA was converted to cDNA using the High Capacity cDNA Reverse Transcription Kit (Applied Biosystems, Waltham, USA). Quantitative RT-PCR was performed using predesigned and customized primers (IDT, Leuven, Belgium, **Supplementary Table 1**) and TaqMan Fast Universal PCR Master Mix (Applied Biosystems) on an ABI Prism 7,500 Sequence Detection System (Applied Biosystems). The relative mRNA expression was determined as 2^-ΔΔCt^, normalized to the mean 2^-CT^ value of the healthy/asymptomatic control and to the 2^-CT^ value of the 18S housekeeping gene.

### 2.8 ^13^C-glucose tracing

250,000 PBMCs were seeded in duplicate at a concentration of 1x10^6^ cells per ml in a 96-well plate in glucose-free RPMI-1640 (Caisson Labs), supplemented with 2 g/L sodium bicarbonate, 2 mM L-glutamine (Gibco), 2% dialyzed FCS (Gibco) and 1 g/L [U-^13^C]-Glucose (Cambridge isotope laboratories, Inc., Tewksbury, MA, USA). To one well, 150 nM hydrocortisone (Sigma-Aldrich) was added. After 24 h of incubation at 5% CO2 and 37°C, a steady state of metabolic ^13^C-labeling was reached (as determined in a preliminary experiment) and the cells were transferred to an Eppendorf tube. The well was washed with ice-cold 0.9% NaCl and added to the tube for centrifugation at 300 g for 5 min. 25 µl of ice-cold extraction buffer (80% methanol) was added to the well to lyse the adherent cells and the plate was kept on ice. The cell pellet was washed with ice-cold 0.9% NaCl without resuspending and lysed with 50 µl of ice-cold extraction buffer. The extracts were pooled and stored at -80°C. The extract was then centrifuged at 20,000 g for 15 min at 4°C. The supernatant was used for metabolite analysis. The pellet was dried in a vacuum concentrator and dissolved in 200 mM NaOH by heating at 95°C for 20 min. After centrifugation for 10 min at 2,650 g, the protein concentration was determined in the supernatant by Bradford assay (Bio-Rad, Hercules, CA, USA).

The metabolites in the methanol-water supernatant were analyzed *via* mass spectrometry using Dionex UltiMate 3,000 LC System (Thermo Scientific) coupled to a Q Exactive Orbitrap mass spectrometer (Thermo Scientific) operated in negative mode. 10 μl of the extract was injected onto a Poroshell 120 HILIC-Z PEEK Column (Agilent InfinityLab). A linear gradient was carried out starting with 90% solvent A (acetonitrile) and 10% solvent B (10 mM Na-acetate in mqH2O, pH 9,3). From 2 to 12 min the gradient changed to 60% B. The gradient was kept on 60% B for 3 minutes and followed by a decrease to 10% B. The chromatography was stopped at 25 min. The flow was kept constant at 0.25 ml/min. The columns temperature was kept constant at 25°C. The mass spectrometer operated in full scan (m/z range [70.0000-1050.0000]) and negative mode using a spray voltage of 2.8 kV, capillary temperature of 320°C, sheath gas at 45 units, auxiliary gas at 10 units. AGC target was set at 3.0E+006 using a resolution of 70,000. Data collection was performed using the Xcalibur software (Thermo Scientific). The data analyses were performed by integrating the peak areas (El-Maven – Polly - Elucidata).

Only metabolites of which ≥20% of all sample (n=109) abundancies were above the limit of detection (mean blank + 3*SD) were included for further analysis. The metabolite abundancies were normalized for protein concentration and expressed relative to the mean of the AC vehicle group. For the ^13^C-incorporation, metabolites with a median fractional contribution of <2% were excluded and the corrected isotopologues with a median <2% were excluded.

### 2.9 Glucose uptake assay and flow cytometry

PBMCs were first starved for 30 min at room temperature, at a concentration of 1x10^6^ cells per ml in glucose-free RPMI-1640 (Caisson Labs), supplemented with 2 g/L sodium bicarbonate, 2 mM L-glutamine (Gibco) and 2% dialyzed FCS (Gibco) in 48 well plates with 500,000 cells per well. Next, 50 µM of 2-(N-(7-Nitrobenz-2-oxa-1,3-diazol-4-yl)Amino)-2-Deoxyglucose (2-NBDG; Thermo Fisher Scientific, Eugene, Oregon, US) was added and uptake was allowed for another 30 min. Suspension cells were then transferred to a FACS tube and the wells were washed with 1 ml of cold FACS buffer (DPBS with 2% FCS and 2 mM EDTA). Remaining cells were detached by 15 min incubation on ice, followed by gentle scraping. After washing of all cells, cells were incubated with Fc-receptor blocking reagent (Miltenyi Biotec, Leiden, The Netherlands) in DPBS at room temperature. Cells were washed and incubated on ice for 25 min with 2 panels of monoclonal antibodies. Both panels contained anti-CD69 (BV421, FN50, BD Biosciences) and anti-GLUT1 (Alexa Fluor 647, 202915, BD Biosciences). One panel contained in addition anti-CD56 (BV510, NCAM16.2, BD Biosciences), anti-CD16 (PE-Cy7, eBioCB16, eBioscience) and anti-CD14 (APC-Cy7, MφP9, BD Biosciences). The other panel contained in addition anti-CD4 (PE-Cy7, A161A1, Biolegend) and anti-CD8 (APC-Cy7, RPA-T8, BD Biosciences). Cells were washed twice and analyzed immediately with a 3-laser FACS Canto II (BD Biosciences). Data were analyzed with FlowJo v10 software (FlowJo LLC, Ashland, OR, USA). The gating strategies of the lymphoid and myeloid cell populations are shown in **Supplementary Figure 1**.

### 2.10 Statistical analysis

The number of data points is indicated in each Figure legend. The GraphPad Prism Software (GraphPad Software, San Diego, CA) was used for most analyses. P-values for the differences in UM versus AC, SM versus AC and SM versus UM groups were calculated by the non-parametric two-tailed Mann-Whitney U-test for quantitative variables and by the non-parametric two-tailed Chi-square test for categorical patient characteristics in **Table 1**. P-values for the analysis of paired data comparing hydrocortisone with vehicle conditions were calculated by the non-parametric Wilcoxon signed-rank test. Bonferroni Holm correction for multiple comparisons was performed on all tests. Non-parametric two-tailed Spearman correlations were computed.

**Table 1:** Patient characteristics.

	AC (N = 23)	UM (N = 21)	SM (N = 26)
Male sex – no. (%)	6 (26)	16 (76) **	15 (58)
Age (years) – median (range)	6.3 (2.5 - 13.3)	6.0 (1 - 15)	5.0 (0.5 - 17)
Severe malnutrition (Z-score<-3 SD) – no. (%)	1 (4)	2 (10)	3 (12)
Systolic blood pressure – median z-score (IQR)	0.2 (-1.1 - 1.8)	1 (-0.1 - 1.7)	0.1 (-1.1 - 0.9)
Diastolic blood pressure – median z-score (IQR)	0.6 (-0.2 - 1.8)	1.3 (0.3 - 2.2)	0.9 (-0.8 - 1.6)
Temperature (°C) – median (IQR)	36.2 (36 - 36.4)	38.2 (36.9 - 39.4) ***	38.8 (37.5 - 40) ***
White blood cells x 10^3^/µl blood – median (IQR)	6.2 (4.8 - 7.5)	9.1 (6.4 - 14.8) **	8.9 (6.5 - 13.8) **
Patients presenting with SM features – no. (%)			
Convulsions	0	1 (5)¥	4 (15)
Respiratory distress	0	0	3 (12)
Prostration	0	0	18 (69)
Coma (Blantyre score<3 or Glasgow score<10)	0	0	0
Severe anemia (Hb< 5 g/dl)	0	0	5 (19)
Hyperparasitemia (> 250,000 parasites/µl)	0	0	9 (35)

Groups were statistically compared for sex and malnutrition by Chi-square test and for age, blood pressure, temperature and white blood cells by Man Whitney-U test. Stars represent the significance level compared to the AC group. No significant differences were found between SM and UM. ¥ less than 2 convulsions within 24h; AC, asymptomatic controls; SM, severe malaria; UM, uncomplicated malaria.

For the metabolomics data, differential metabolite abundancies and ^13^C labeling between the vehicle condition of AC, UM and SM groups were screened by the Partial Least Squares Method-Discriminant Analysis (PLS-DA) and Variable Importance in Projection (VIP) values with MetaboAnalyst v5.0 and by the Student’s t-test using Microsoft Excel. Paired GC effects, expressed as the log2 fold changes were analyzed by PLS-DA with MetaboAnalyst and were analyzed with the paired Student’s t-test in Microsoft Excel. Log2 fold changes by hydrocortisone of specific metabolites were furthermore compared between AC, UM and SM groups by the use of the non-parametric two-tailed Mann-Whitney U-test in GraphPad Prism. Bonferroni Holm corrections were performed on 3 comparisons made by the unpaired t test, the paired t test and Mann-Whitney U-test. Donuts and histogram plots of the metabolomics data were generated in Python. In all the figures, only significant differences are shown with * p< 0.05, ** p< 0.01, *** p< 0.001, ****p< 0.0001.

## 3 Results

### 3.1 Characteristics of the cohort

**Figure 1:**
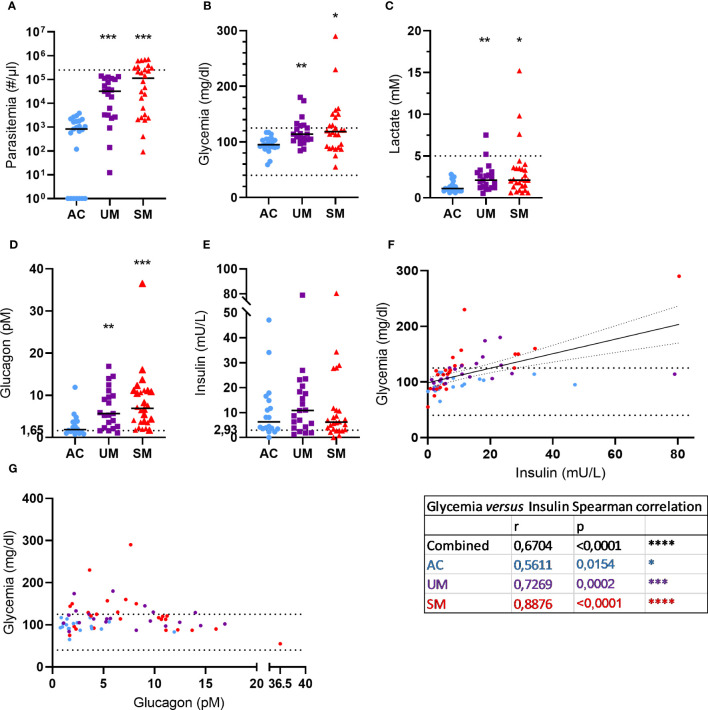
Blood and plasma analytes. **(A)** Parasite levels were microscopically determined on thick smears and calculated using the concentration of white blood cells in the blood that was determined. The dotted line represents the hyperparasitemia threshold of 250,000 par/µl. **(B, C)** Glucose and lactate levels were measured in whole blood. The dotted lines show the hypo- and/or hyper- thresholds. **(D, E)** Plasma glucagon and insulin levels. Dotted lines represent the limits of detection. **(F, G)** Correlation between insulin or glucagon levels and glycemia. Spearman r- and p-values are shown. **(A, G)** Each symbol represents data from an individual. **(A, E)** Horizontal lines in between data points represent group medians and analysis was by Mann-Whitney U-test. Asterisks above individual data sets indicate statistical differences compared to the AC group. Only significant differences are indicated. * p<0.05, ** p<0.01, *** p<0.001, **** p<0.0001. AC n=23 **(A-C)** n=18 **(D-G)**; UM n=21; SM n=26.

A total of 70 children were included in the study, of which 23 were included in 2019 and 47 in 2021. The characteristics of the 3 study groups are summarized in **Table 1**. **Figure 1** shows the levels of several blood and plasma analytes. Parasitemia was similarly increased in UM and SM compared to AC (**Figure 1A**). 35% of patients with SM had hyperparasitemia (**Table 1**). None of the AC showed symptoms but 70% appeared asymptomatic parasite carriers as detected by microscopy, which could be expected due to the high transmission intensity.

### 3.2 Patients with clinical malaria have higher glucose and lactate levels alongside increased glucagon

Metabolic disturbances such as lactic acidosis and hypoglycemia are common in malaria and contribute to the severity of disease. In our patient population, 29% UM and 35% of SM patients showed hyperglycemia (>125 mg/dl), which can also occur after malaria infection, and no hypoglycemia (< 40 mg/dl) was observed (**Figure 1B**). Furthermore, 4 cases of hyperlactatemia were detected amongst the patients and lactate levels were increased in UM and SM compared to AC (**Figure 1C**).

Glycemia is tightly regulated by the pancreatic hormones, insulin and glucagon. Plasma glucagon levels (**Figure 1D**) increased in UM and SM compared to AC, whereas insulin levels (**Figure 1E**) were similar between the groups. In all children, insulin levels correlated with glycemia (**Figure 1F**) with the strongest correlation in the SM group specifically. In contrast, glucagon levels did not correlate with glycemia (**Figure 1G**). In fact, almost all patients with high glucagon levels (>10 pM) had normoglycemia, whereas less increased glucagon levels (<10 pM) coincided with hyperglycemia in some patients.

### 3.3 PBMCs from patients with UM and SM are activated but do not increase their glucose uptake

**Figure 2:**
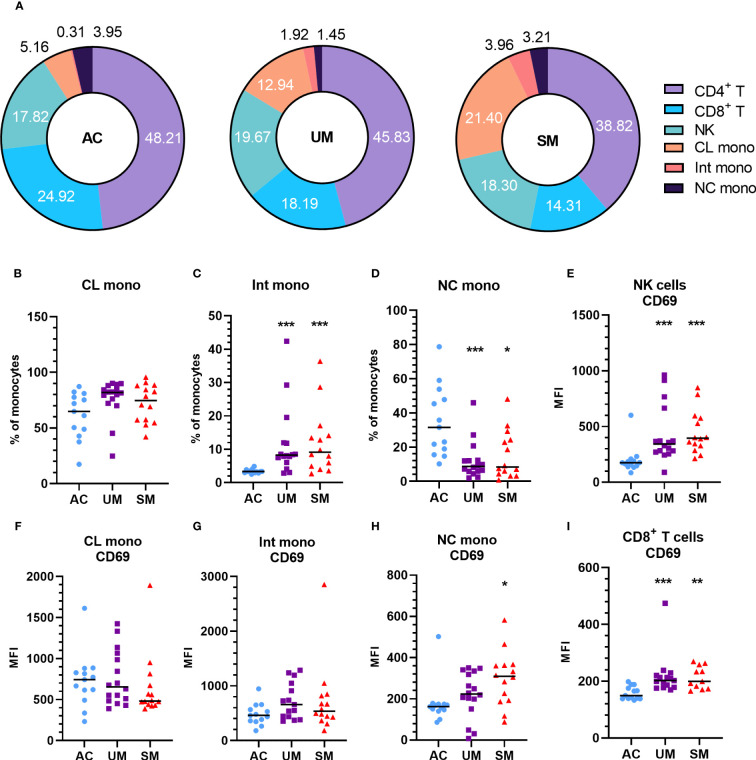
Increased monocytes and activation of PBMC subsets in patients with malaria. PBMCs were isolated and flow cytometry was performed on fresh cells. **(A)** Percentages of different cell subsets in the AC, UM and SM group. **(B-D)** Percentage of monocyte subsets relative to the total number of monocytes. **(E-I)** Median fluorescence intensity (MFI) of CD69 on the respective cell subsets. **(B-I)** Each symbol represents data from an individual. Horizontal lines in between data points represent group medians and analysis was by Mann-Whitney U-test. Asterisks above individual data sets indicate statistical differences compared to the AC group. * p<0.05, ** p<0.01, *** p<0.001. NK, Natural Killer; CL mono, classical monocyte; Int mono, Intermediate monocyte; NC mono, Non-classical monocyte. Only significant differences are indicated. AC n=13; UM n=16; SM n=14. For CD4^+^ T cells **(A)** UM n=15; SM n=13 and for CD8^+^ T cells **(A, G)** UM n=15 SM n=11.

PBMCs were isolated and analyzed by flow cytometry. The percentage of monocytes increased in UM and SM compared to AC at the expense of CD4^+^ and CD8^+^ T cells (**Figure 2A**). In the patients, monocytes were more inflammatory, since the population mainly consisted of classical monocytes and increased percentages of intermediate monocytes, alongside reduced proportions of non-classical monocytes (**Figures 2B-D**). Moreover, the non-classical monocytes, but not intermediate and classical monocytes, of SM patients expressed higher levels of the activation marker CD69 compared to AC. Also CD8^+^ T cells and NK cells expressed more CD69 in patients compared to AC (**Figures 2E–I** **)**. GLUT1, the ubiquitously expressed glucose transporter, is known to increase upon activation of immune cells (22). However, despite the activated state of the immune cells in the patients with clinical malaria compared to AC, GLUT1 surface expression was similar or even decreased in the different cell subsets (**Supplementary Figure 2A**). Furthermore, the uptake of the fluorescently labelled glucose derivative, 2-NBDG, was reduced in CD4^+^ and CD8^+^ T cells of patients compared to AC, in line with lower GLUT1 expression. Of all the cell types, only the intermediate monocytes showed higher uptake of 2-NBDG in the patients compared to AC (**Supplementary Figure 2B**).

### 3.4 Glucocorticoids increase in patients with clinical malaria in spite of heterogenous ACTH levels

**Figure 3:**
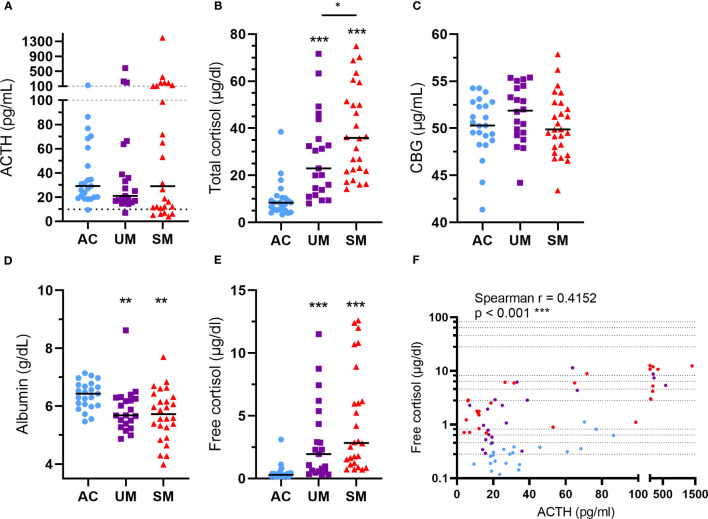
Alterations in HPA axis hormones and proteins. Plasma concentrations of ACTH **(A)**, total cortisol **(B)**, CBG **(C)**, albumin **(D)** and free cortisol **(E)**. **(A)** The dotted line represents the threshold for low ACTH levels of 10 pg/ml. **(A-F)** Each symbol represents data from an individual. **(A-E)** Horizontal lines in between data points represent group medians and analysis was by Mann-Whitney U-test. Asterisks above individual data sets indicate statistical differences compared to the AC group. Horizontal lines with asterisks on top indicate the levels of statistical significance between the indicated groups.* p<0.05, ** p<0.01, *** p<0.001. **(F)** Correlation between free cortisol and ACTH. Spearman r- and p-values are shown. Only significant differences are indicated. AC n=23; UM n=21; SM n=26.

ACTH levels were not different between patients and AC but showed a higher spread in the SM group (**Figure 3A**) with some having high ACTH levels and others having relatively low ACTH levels compared to the AC group. Total cortisol levels increased in patients versus AC with a higher increase in SM compared to UM (**Figure 3B**). The bioavailability of cortisol in plasma is determined by the saturable binding to CBG and the non-saturable binding to albumin. Only the unbound or ‘free’ cortisol fraction is able to diffuse across the cell membrane to bind to the GR. Therefore, plasma CBG and albumin concentrations were determined. CBG levels were similar between groups (**Figure 3C**) and albumin levels were decreased in patients with UM and SM compared to AC (**Figure 3D**). The calculated free cortisol levels were higher in patients than AC (**Figure 3E**). Both total and free cortisol were thus elevated upon malaria disease with higher total cortisol levels in SM compared to UM. In all patients with malaria, total cortisol levels correlated with parasitemia (Spearman r = 0.41, p = 0.004), glucagon (Spearman r = 0.30, p = 0.038) and lactate levels (Spearman r = 0.3140, p = 0.0316), suggesting a link between cortisol responses and other malaria-associated disturbances. Cortisol did not always correlate with ACTH, the main inducer of cortisol production and release. Some patients had increased free cortisol levels together with suppressed ACTH levels (**Figure 3F**). This dissociation between ACTH and cortisol in a selected subset of patients might be explained by effective feedback inhibition by cortisol on ACTH production in combination with decreased cortisol breakdown.

### 3.5 PBMCs from patients with clinical malaria are less sensitive to GC induced gene expression

**Figure 4:**
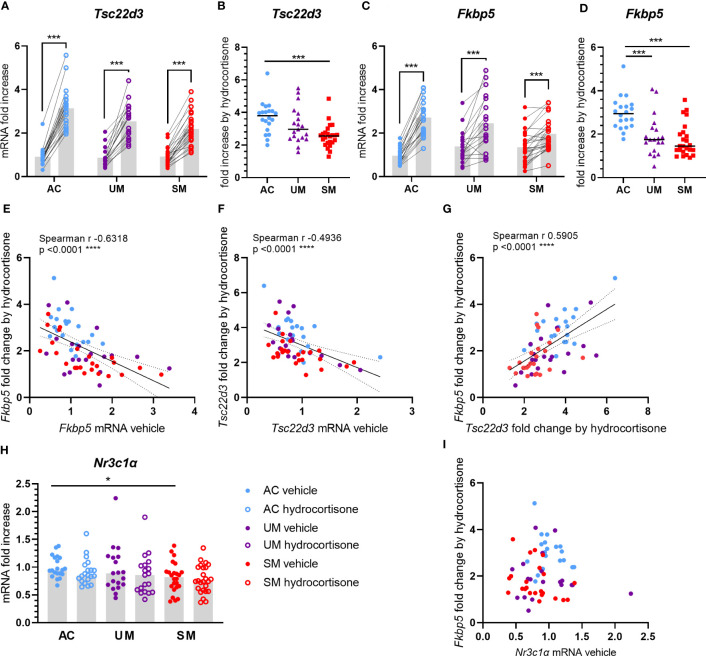
Induction of GILZ and FKBP5 transcripts by hydrocortisone is reduced in PBMCs from patients. PBMCs were isolated and incubated for 2 hours with vehicle or 150 nM hydrocortisone. mRNA was extracted and levels of *Tsc22d3* (GILZ), *Fkbp5* and *Nr3c1α* (GRα) were measured by qRT-PCR. **(A, C)** The mRNA fold increase was calculated compared to the mean of the AC vehicle group and the paired effect of the hydrocortisone *versus* vehicle condition was analyzed for the controls and patients. **(B, D)** Fold increase by hydrocortisone defined by the ratio of the mRNA fold increase of the hydrocortisone condition over the vehicle condition. **(E-G, I)** Correlation analyses by Spearman test. **(H)** mRNA fold increase compared to the AC vehicle group. **(A-I)** Each symbol represents data from an individual. Analysis was by Mann-Whitney U-test or Wilcoxon test for the paired data. Only significant differences are indicated. * p<0.05, *** p<0.001, **** p<0.0001. AC n=20; UM n=19; SM n=24.

Upon cytoplasmic binding of GCs to the GR, the GC-GR complex dissociates from chaperones and translocates to the nucleus where it activates or represses a wide range of genes. To assess whether the sensitivity of PBMCs to GCs was affected by malaria, mRNA expression of two genes that are classically induced by GCs were measured in cells treated with 150 nM of hydrocortisone *versus* unstimulated cells. *Tsc22d3* encodes glucocorticoid induced leucine zipper (GILZ), a protein with various anti-inflammatory and immunosuppressive properties. Having clinical malaria did not alter the basal mRNA expression of GILZ in PBMCs (**Figure 4A**). Transcripts of GILZ were efficiently induced by hydrocortisone in the three groups (**Figure 4A**), but the fold increases in patients with SM were clearly reduced (**Figure 4B**). FK506-binding protein 5 (FKBP5) is a co-chaperone protein that lowers the binding affinity of the GR to GCs. Similar to the results with *Tsc22d3*, though hydrocortisone could induce FKBP5 mRNA in all groups, the induction appeared deranged in the UM and SM group (**Figure 4C**), leading to a marked reduction in fold increase in both UM and SM patients compared to AC (**Figure 4D**). Despite no difference in basal mRNA levels of FKBP5 between groups (**Figure 4C**), more variability was observed in the patients. Importantly, the basal expression of FKBP5 and GILZ correlated negatively with the fold increase of the respective expression by the *ex vivo* added hydrocortisone (**Figures 4E, F** **)**. The FKBP5 fold induction by hydrocortisone also strongly correlated with the GILZ fold induction (**Figure 4G**). Only PBMCs from patients with SM showed a mild reduction in *Nr3c1*, encoding GRα (**Figure 4H**). GC responsiveness, reflected by the FKBP5 fold induction, did not correlate with the expression of GRα (**Figure 4I**), which suggests that reduced GRα expression is not causing the GC insensitivity in PBMCs from patients with clinical malaria. Altogether, these data indicate that PBMCs of patients with clinical malaria, especially with SM, respond less to GCs.

### 3.6 Malaria infection in mice cause reduced corticosterone sensitivity of splenocytes

**Figure 5:**
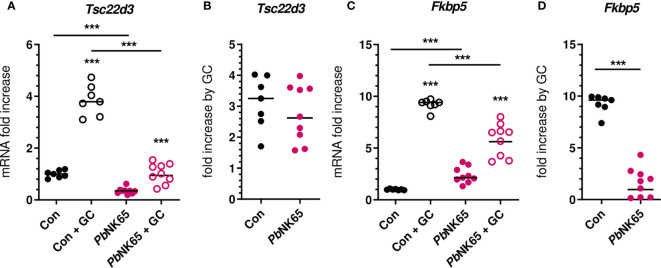
Malaria infection in mice causes reduced corticosterone sensitivity of splenocytes. Splenocytes were isolated from spleens of non-infected control (Con) C57Bl/6J mice and *Plasmodium berghei* NK65 (*Pb*NK65)-infected C57Bl/6J mice (day 9 post-infection) and stimulated for 2 hours without or with 400 nM of corticosterone (GC). mRNA levels of *Tsc22d3* (GILZ) and *Fkbp5* were measured by qRT-PCR. **(A, C)** mRNA fold increase compared to the Con group. **(B, D)** Fold increase by GC defined by the ratio of the mRNA fold increase of the GC condition over the untreated condition. The data originates from two separate experiments. Each symbol represents data from an individual mouse. Horizontal lines in between data points represent group medians. Analysis was by Mann-Whitney U-test. Horizontal lines with asterisks on top indicate the levels of statistical significance between the indicated groups. Asterisks above individual data sets indicate the levels of statistical significance compared to the unstimulated group. Only significant differences are indicated. *** p<0.001. Con n=7; *Pb*NK65 n=9.

Mouse models of malaria represent excellent tools to study the pathophysiology of malaria complications and were used here to confirm the GC insensitivity that was observed in PBMCs from patients. C57BL/6J mice were infected with *Pb*NK65 parasites, resulting in experimental MA-ARDS. GC sensitivity was compared between non-infected control mice and severely ill *Pb*NK65-infected mice by measuring the induction of mRNA expression of GILZ and FKBP5 after stimulation of splenocytes with corticosterone (**Figure 5**). GILZ expression was potently suppressed in infected mice. Both baseline and corticosterone-induced mRNA expression of GILZ were reduced in the splenocytes of infected mice compared to control mice (**Figure 5A**), despite similar fold increases upon corticosterone stimulation (**Figure 5B**). FKBP5 levels were significantly increased in splenocytes from infected mice compared to control mice (**Figure 5C**). Furthermore and analogously to our patient data (**Figure 4D**), the FKBP5 mRNA expression was lower after corticosterone stimulation of splenocytes of infected mice compared to control mice (**Figure 5C**). As a result, FKBP5 levels showed a drastic reduction in fold increase after corticosterone stimulation (**Figure 5D**). Therefore, SM in mice results in decreased capacity for target gene transcription in immune cells by GCs.

### 3.7 GC-mediated induction of cAMP and AMP is reduced in PBMCs from patients with clinical malaria

To assess whether PMBCs from malaria patients showed, besides transcriptional, also altered metabolic responses to GCs, *[U-^13^C]-glucose* tracer metabolomics analysis was performed on PBMCs incubated with or without hydrocortisone. Metabolite abundancy and ^13^C incorporation in more than 40 intermediates of different metabolic pathways including glycolysis, Krebs cycle, nucleotide synthesis, redox metabolism and pentose phosphate pathway (PPP) were analyzed to investigate how glucose is metabolized by PMBCs.

**Figure 6:**
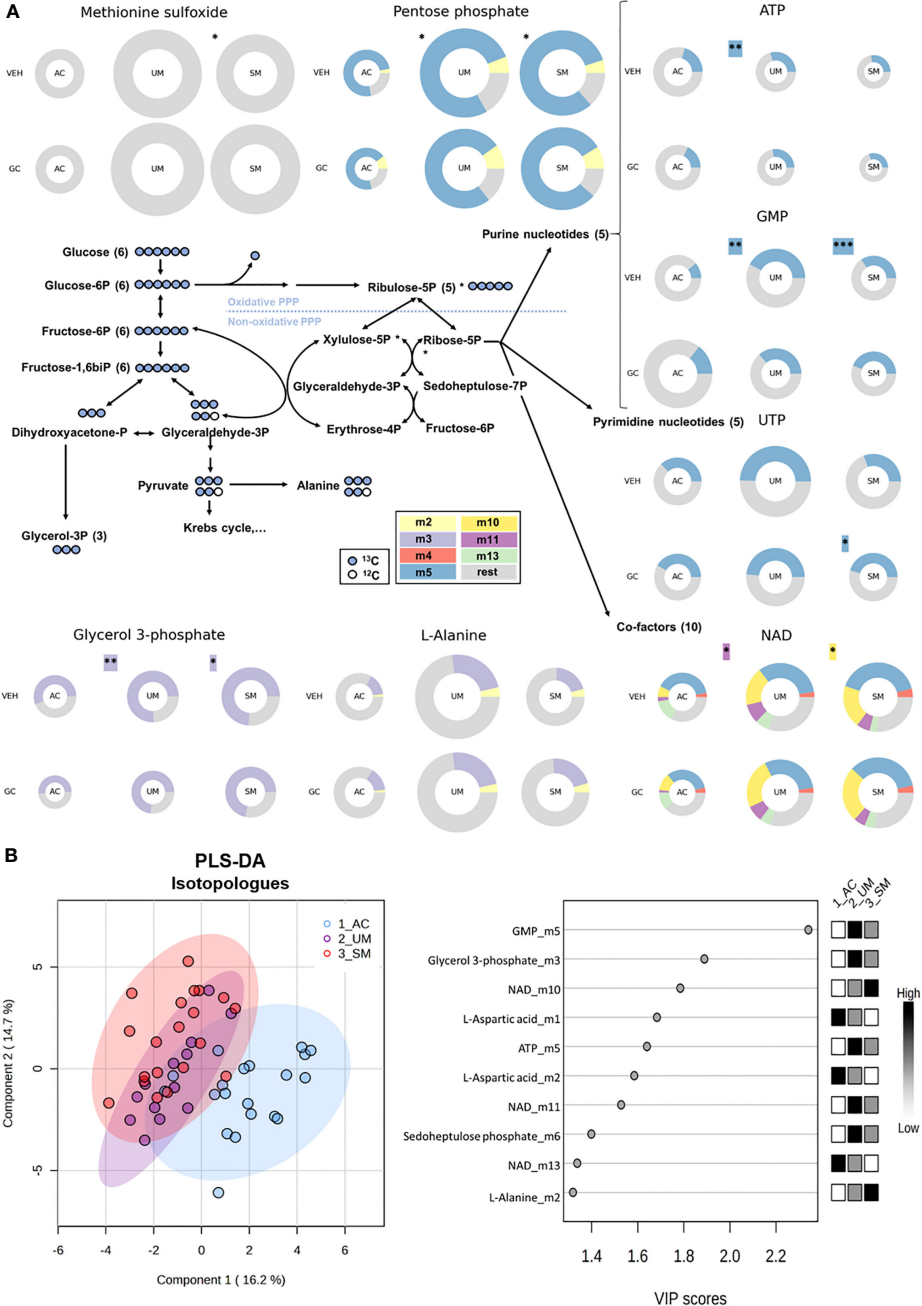
The pentose phosphate pathway is enhanced in PBMCs from patients with malaria. PBMCs were isolated and incubated for 24 hours with [U-^13^C]-glucose tracer and vehicle (VEH) or 150 nM hydrocortisone (GC). Metabolic extracts were analyzed *via* mass spectrometry. **(A)** Schematic visualization of the pentose phosphate pathway with donut plots of selected metabolites. The size of the donuts represents the average relative abundancy compared to the AC VEH condition. The average relative contribution of an isotopologue is represented by the different colors as defined in the legend. The rest fraction contains the unlabeled and rare isotopologue (<2%) fractions. Analysis was done by t-test for UM and SM *versus* AC as indicated with asterisks next to the donuts on the VEH row. A paired t-test for the GC effects in AC, UM and SM is indicated with asterisks next to the donuts on the GC row. Non-highlighted asterisks represent effects in abundancies. Highlighted asterisks correspond with effects in specific isotopologues. Only significant differences are indicated. **(B)** PLS-DA was performed to separate AC, UM and SM based on 41 corrected isotopologues from VEH-incubated PBMCs. PLS-DA: Partial Least Squares Method-Discriminant Analysis; PPP, pentose phosphate pathway; VIP, Variable Importance in Projection. * p<0.05; ** p<0.01; *** p<0.001. Abundancies: AC n=20; UM n=14 (12 paired); SM n=22 veh (21 paired). Isotopologues: AC n=20; UM n=13 (11 paired); SM n=20 (18 paired).

We first determined differences between PBMCs of AC, UM and SM, incubated without hydrocortisone. Significant changes (t test p<0.05 and fold change >2) in glucometabolic pathways are highlighted with asterisks in **Figure 6A** and **Supplementary Figure 3** shows the respective graphs for the individual metabolites. PBMCs of SM patients showed a higher abundancy of methionine sulfoxide, suggesting increased oxidative stress. Furthermore, the abundancy of pentose phosphate was increased in UM and SM patients compared to AC. The percentages of specific isotopologues provide information on the relative contribution of glucose to a certain metabolite through various metabolic routes. Specific isotopologues with 1, 2, 3,… labelled carbons are, respectively, named m1, m2, m3. A PLS-DA analysis with 41 corrected isotopologues indicated that ^13^C incorporation was clearly different in PBMCs from AC *versus* UM and SM patients with the most distinctive isotopologues listed in **Figure 6B**. In addition to increased levels of pentose phosphate, enhanced PPP metabolism in UM and/or SM patients with malaria is shown by an increased m5 (or ribose) labeling in purine nucleotides ATP and GMP and increased m10 and m11 labeling in co-factor NAD^+^, both end products from the non-oxidative part of the PPP (**Figure 6A**; **Supplementary Figure 3**). This demonstrates that upon clinical malaria, carbons of nucleotides and co-factors in PBMCs, incubated *ex vivo*, are increasingly derived from glucose. When the PPP merges with glycolysis through glyceraldehyde-3-phosphate, an m2 instead of m3 labeling could be expected. In L-alanine, which is generated *via* pyruvate, a trend towards more m2 labeling is observed in PBMCs from SM patients (corrected p = 0.05114). Furthermore, increased m3 labeling is found in glycerol 3-phosphate in patients with clinical malaria versus AC. Most Krebs cycle intermediates were excluded in the analysis due to either low abundancies and/or low fractional contribution percentages.

**Figure 7:**
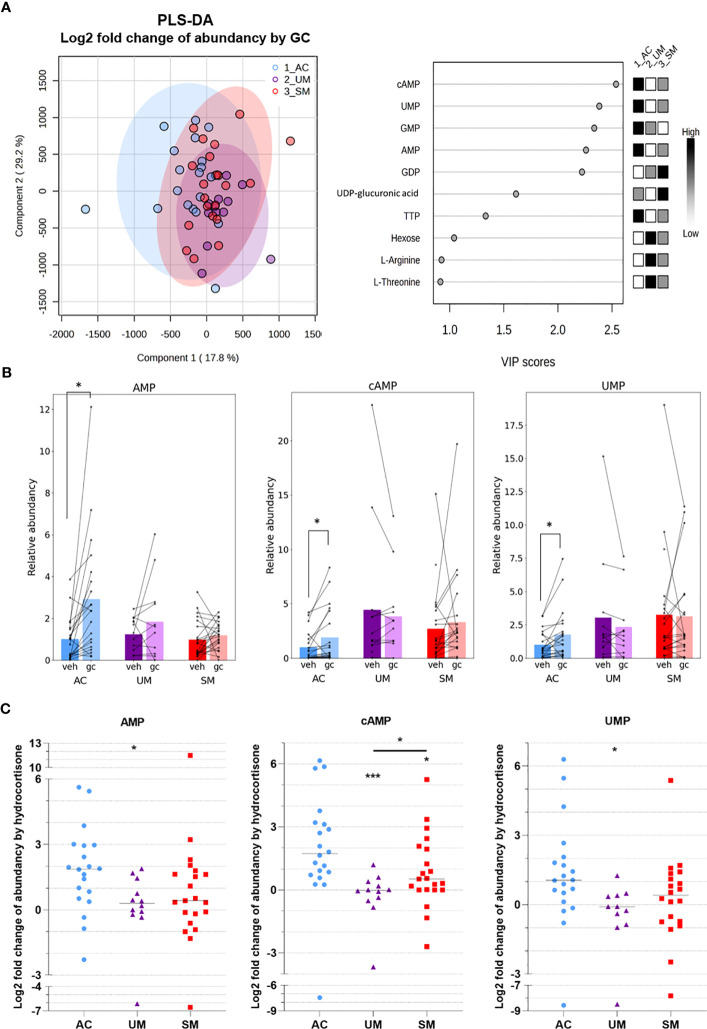
GC-mediated induction of cAMP and AMP is reduced in PBMCs from patients with malaria. PBMCs were isolated and incubated for 24 hours with [U-^13^C]-glucose tracer and vehicle (VEH) or 150 nM hydrocortisone (GC). Metabolic extracts were analyzed *via* mass spectrometry. **(A)** GC-induced Log2 fold changes in abundancies of 47 metabolites were analyzed by PLS-DA. **(B)** Analysis of the GC versus VEH condition for the relative abundancy of AMP, cAMP and UMP by paired t-test. **(C)** Log2 fold increase of abundancy by hydrocortisone. Comparison between groups by Mann-Whitney U-test. Each symbol represents data from an individual. Bars in **(B)** represent the mean, lines in **(C)** represent the median. Asterisks above individual data sets indicate statistical differences compared to the AC group. Only significant differences are indicated. PLS-DA, Partial Least Squares Method-Discriminant Analysis; VIP, Variable Importance in Projection. AC n=20; UM n=11-12; SM n=20-21. * p<0.05, *** p<0.001.

To investigate the metabolic response to GCs, metabolite abundancies and labeling were compared between unstimulated (vehicle) cells and cells stimulated with a physiological concentration of hydrocortisone. A PLS-DA analysis based on the log2 fold change in abundancies of 47 metabolites indicated that AC, UM and SM patients mainly differed in their response of the levels of several nucleotides to GC stimulation (**Figure 7A**). Indeed, paired analysis showed that hydrocortisone increased the abundancy of AMP, cAMP and UMP in the AC, but not in PMBCs from patients with clinical malaria (**Figure 7B**). The fold increase of AMP and UMP was lower in UM patients compared to AC and the fold increase of cAMP was reduced in both UM and SM patients compared to AC (**Figure 7C**). This nucleotide response was not reduced in SM compared to UM. cAMP increases are even higher in SM *versus* UM patients. Furthermore, GCs increased m5 labeling of UTP in SM (**Figure 6A**; **Supplementary Figure 3**).

Altogether, PBMCs from patients with clinical malaria showed increased activity of the PPP, which was not affected by GC stimulation. Importantly, reduced cAMP induction after GC stimulation compared to ACs was observed. Non-genomic GC signaling might thus be impaired in PBMCs of malaria patients. These data further stress that upon malaria disease, GC insensitivity develops as measured in a versatile way both on a transcriptional and signaling level.

## 4 Discussion

In children living in Cameroon, cortisol levels and the sensitivity of PBMCs to hydrocortisone were compared between AC, UM and SM. Glucagon and cortisol levels were considerably increased in patients with clinical malaria, alongside higher lactate and glucose levels. This was also found in other studies (23–26). ACTH levels were highly variable with low and high extremes in some patients. Isolated PBMCs from clinical malaria patients contained more monocytes and activated cells, and showed increased pentose phosphate pathway activity, resulting in more *de novo* production of nucleotides and co-factors from glucose. Importantly, the induction of GILZ and FKBP5 expression by hydrocortisone was less efficient in PBMCs from diseased patients compared to AC. Similarly, hydrocortisone was also less able to increase the levels of cAMP in PBMCs of patients with clinical malaria, indicating that sensitivity of PBMC to both genomic and non-genomic effects of GCs decreases during clinical malaria.

Despite the limitations of this study, the clear findings justify new investigations on GC dysregulation as a contributor to malaria disease severity. Limitations are the modest sample size with a relatively large spread in age, differences in sex distribution, and the high proportion of asymptomatic parasite carriers among the children in the control group. Also, no CM cases or patients with severe hypoglycemia were included. We hypothesize that GC sensitivity might even further decrease in these severe complications of malaria, but this needs further investigation. Overall, SM is typically a highly heterogeneous condition, which may also explain part of the variability in the data of our study. Further investigations are needed to determine whether glucocorticoid insensitivity is present in the different complications.

The timing since the onset of disease was not registered, but it was probably variable between patients within our cohort. This might affect the relationship between ACTH and cortisol levels, as described during critical illness (27). During the initial acute phase of critical illness, an ACTH-driven rise in cortisol is initiated. In a later phase, a negative feedback on the hypothalamus and anterior pituitary takes place, with a combination of high cortisol levels and suppressed ACTH levels. The latter was observed for some SM patients in our study. Importantly, some old studies investigated the responsiveness of the HPA axis after infection with *P. falciparum* through suppression and activation tests, as reviewed previously (7). In UM, the HPA axis was found to be intact but in SM, the pituitary secretion of ACTH was suboptimal (28, 29). In addition to the ACTH-driven release of cortisol, reduced enzymatic breakdown of cortisol in liver and kidney might increase circulatory levels of cortisol. Davis et al. found that in adults with severe malaria, the cortisol elimination rate was lower (4). In critically ill children, cortisol breakdown is reduced by a decreased activity of 11β-hydroxysteroid-dehydrogenase-2 and the A-ring reductases, 5α-reductase and 5β-reductase (30). Testing cortisol metabolites in the urine of patients with malaria might be useful to estimate whether a reduced activity of cortisol metabolizing enzymes contributes to increased cortisol levels upon malaria infection. Our results also show a high variability in cortisol levels in the patients with clinical malaria, suggesting that a subset of patients might have insufficient cortisol levels to cope with severe illness.

The cortisol that is released upon infection is believed to be decisive for a range of vital processes such as immune responses, vascular function, and metabolism, which are all crucial in malaria (7). Glucagon and cortisol act synergistically as glucose counterregulatory hormones to increase circulatory glucose under stress or fasting conditions through different ways, including increasing gluconeogenesis and decreasing peripheral glucose uptake (31). In our patient cohort, induced glucagon and cortisol levels are indeed paralleled by increased glucose levels. Van Thien et al. described that in adult cerebral malaria, cortisol levels and glycemia were higher and that glucose production *via* gluconeogenesis was increased (24). Although hyperglycemia is less frequently reported and presumed to be less acutely troublesome in malaria compared to hypoglycemia, it has been associated with SM and more specifically cerebral malaria (32–36). Also, continuous blood monitoring in hospitalized children with malaria identified frequent episodes of both hypo- and hyperglycemia (37), overall suggesting that glucose homeostasis is unstable upon infection with malaria and that the regulation by GCs might be crucial.

Protection against malaria considerably relies on immune-mediated clearance of the parasite, involving extensive phagocytic activity, T lymphocyte activation and antibody production (2). These elicited immune responses might have an impact on the metabolic homeostasis of the host upon infection with *Plasmodium* parasites, because leukocyte activation and proliferation are paralleled by drastic metabolic changes including enhanced aerobic glycolysis and glucose consumption (38, 39). Although PBMCs from the patients contained more activated cells, the uptake of 2-NBDG and the surface expression of the glucose transporter GLUT-1 were not increased. However, metabolomics analysis identified that immune cell activation in patients was accompanied by higher levels of methionine sulfoxide and glycerol-3-phosphate and intensified usage of the pentose phosphate pathway to generate nucleotides and cofactors. These findings are in line with previous studies describing increased m5 labeling in UMP, AMP and GMP of *in vivo* murine T effector cells compared to T naïve cells (40) and decreased m5 labeling of nucleoside phosphates after PD-L1 inhibition of activated human T cells (41). Altogether, these metabolic responses in PBMCs from patients with clinical malaria illustrate the requirement for activated immune cells to survive and proliferate through mounting anti-oxidative responses and producing building blocks such as nucleotides and glycerol-3-phosphate from glucose.

GCs are well known for their anti-inflammatory and immunosuppressive effects, which also seem to play a role in malaria. Abdrabou et al. reported that upon *P. falciparum* infection of children, serum steroids - including corticosteroids - were upregulated and associated with immunosuppression (42). Our results show that circulatory cortisol levels indeed increased in patients with clinical malaria but that sensitivity of patient PBMCs to *ex vivo* hydrocortisone is reduced, since the induction of GILZ and FKBP5 mRNA and cAMP responses were impaired. Besides an *in vivo* dexamethasone suppression test, GC sensitivity is often assessed in PBMCs by evaluating GR number and affinity, dexamethasone-mediated inhibition of proliferation and dexamethasone-mediated transactivation and transrepression of genes (43–45). To estimate the GC sensitivity in this study, physiological cortisol/corticosterone levels rather than supraphysiological levels of a synthetic corticosteroid were applied without prior activation of the cells, and the transactivation of both GILZ and FKBP5, two typical target genes of GCs was used as a readout. Despite the limited sample size, our results suggest that the GC sensitivity is reduced upon clinical malaria disease in our patient cohort. GILZ is an essential mediator of the anti-inflammatory effects of GCs and FKBP5 impacts the conformation and reduces the binding affinity of the GR, corresponding with a negative feedback loop of GCs (46, 47). GCs have rapid non-genomic effects by inducing increased cAMP levels (11). cAMP activates protein kinase A (PKA) and the interaction of PKA with GR plays a role in the GR-mediated inhibition of NF-kB (48). Furthermore, the observed GC-induced increase in AMP is most likely a consequence of the conversion of cAMP to AMP by phosphodiesterases. By analyzing the induction of GILZ, FKBP5 and cAMP levels, our results indicate that the sensitivity to both genomic and non-genomic effects of GCs is decreased in UM and SM patients *versus* asymptomatic controls. This study captures GC responses along a gradient of malaria severity ranging from the AC group that includes asymptomatic parasite carriers to the UM and SM group with higher parasitemia levels. Moreover, comparing GC sensitivity among different malaria complications including CM and severe hypoglycemia might indicate whether GC resistance further aggravates in these specific complications.

GC insensitivity or resistance occurs in several disorders including chronic obstructive pulmonary disease, acute respiratory distress syndrome, asthma, rheumatoid arthritis and sepsis. This resistance impacts the treatment and possibly the pathogenesis of these diseases (13, 49). Different molecular mechanisms of GC resistance have been proposed over the past decades, including mutations in the GR, increased expression of the alternatively spliced variant GRβ, defective GR binding and translocation, chromatin remodeling and cytokine signaling (e.g. IL-2, TNF-a) (49). In this study, we observed that basal FKBP5 expression levels inversely correlated with responsivity to GCs, i.e. the FKBP5 fold change by the exogenous hydrocortisone. In other disorders, FKBP5 was also related to GC responses and resistance. Excessive exposure to cortisol in patients with Cushing syndrome induces high FKBP5 mRNA expression in blood cells, which returned to baseline after successful surgery (50). In asthmatic patients, high FKBP5 expression in airway epithelial cells associated with a poor response to corticosteroid treatment and FKBP5 overexpression in lymphocytes of squirrel monkeys mediated their inborn GC resistance (51, 52). Induction of FKBP5 by the GR might thus reduce GC sensitivity in patients with clinical malaria. Still, various other mechanisms could contribute to the GC insensitivity in patients with malaria and this needs further investigation.

Importantly, reduced GC sensitivity or GC resistance might overall contribute to the severity of malarial disease as illustrated in different mouse models of malaria in which absence of adrenal hormones lead to lethal hypoglycemia and hyperinflammation (16). GC resistance upon malaria infection might also clarify why two clinical trials in the 1980s failed to show benefit from dexamethasone treatment of cerebral malaria, whereafter the interest in GCs in malaria ceased (14, 15). With this study, we want to underscore that more in-depth investigation of GC resistance in different malaria patient subpopulations and the contribution to disease severity is warranted. GC treatment of critical illness-related corticosteroid insufficiency is complex and highly controversial (27). Yet, investigating the detailed GC biology in malaria and define whether or not some specific SM patient subgroups might benefit from GC therapy is worthwhile, since complications of malaria still cause many annual deaths worldwide.

## Data availability statement

The original contributions presented in the study are included in the article/**Supplementary Material**. Further inquiries can be directed to the corresponding author.

## Ethics statement

The studies involving human participants were reviewed and approved by UZ Leuven Ethical Committee (Protocol number S62395) and the National Ethics Committee for Research in Human Health (CNERSH) in Yaoundé, Cameroon (Protocol number 2019/03/1150/CE/CNERSH/SP). Written informed consent to participate in this study was provided by the participants’ legal guardian/next of kin. The animal study was reviewed and approved by the Animal Ethics Committee of the KU Leuven.

## Author contributions

LV, FP, BF, PD, SK, and CD performed the laboratory experiments. CN, JT, and PN executed and oversaw the clinical work. LV analyzed the data. PV, LV, and LA conceived the study. BG helped with the setup and interpretation of the metabolomics part. LV and PV wrote the first drafts of the manuscript. All authors critically read and edited the manuscript. All authors contributed to the article and approved the submitted version.
