# Supplementary material for: Glucocorticoid dysfunction in children with severe malaria
Source: Front Immunol. 2023 Jul 10;14:1187196. doi: 10.3389/fimmu.2023.1187196 (PMC10364055; doi:10.3389/fimmu.2023.1187196)
Supplement: Supplementary file 1 [file DataSheet_1.pdf]

Supplementary Material

1 Supplementary Figures and Tables

Supplementary Table 1. List of all qRT-PCR primers

Predesigned qPCR assays (IDT)			
**Name**	**Ref Seq**	**Exon location**	**Assay ID**
Human			
18S	NR_003286(1)	Exon 1-1	Hs.PT.39a.22214856.g
TSC22D3 (GILZ)	NM_001015881(3)	Exon 4-5	Hs.PT.58.4331913
FKBP5	NM_001145775(4)	Exon 7-8	Hs.PT.58.20699604
Mouse			
TSC22D3 (GILZ)	NM_001077364(1)	Exon 2-5	Mm.PT.58.42845144
FKBP5	NM_010220(1)	Exon 8-9	Mm.PT.58.45861921

Customized primers			
**Name**	**Forward primer**	**Reverse primer**	**6-FAM/ZEN/IBFQ Probe**
GRα	5’-AGAACTGGCAGCGGTTT-3’	5’-GATTGGTGATGATTTCAGCTAAC-3’	5’-ACTCTTGGATTCTATGCATGAAGTGGTTGA-3’

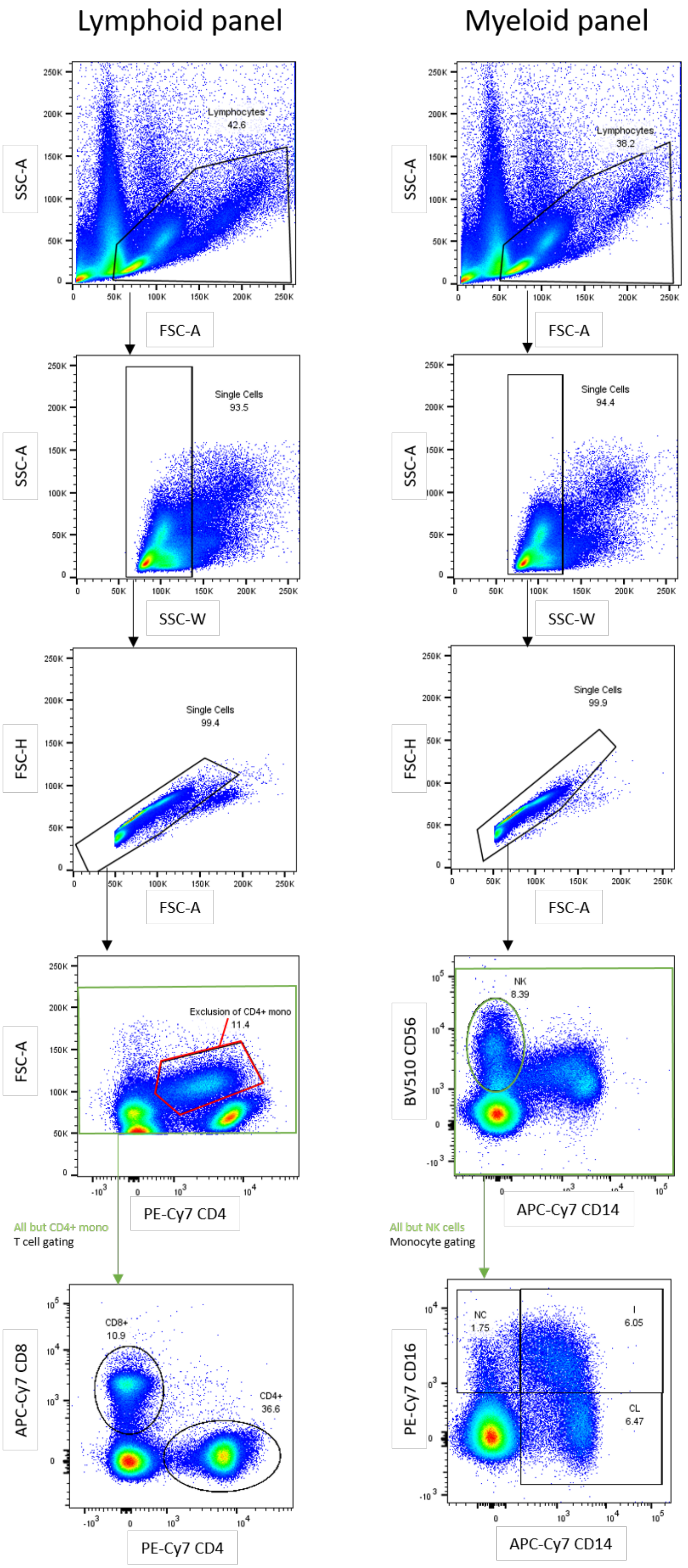

Lymphoid panel
Myeloid panel
Lymphocytes
42.6
Lymphocytes
38.2
SSC-A
FSC-A
Single Cells
93.5
Single Cells
94.4
SSC-W
Single Cells
99.4
Single Cells
99.9
FSC-H
Exclusion of CD4+ mono
11.4
NK
8.39
BV510 CD56
APC-Cy7 CD14
PE-Cy7 CD4
All but CD4+ mono
T cell gating
All but NK cells
Monocyte gating
CD8+
10.9
CD4+
36.6
APC-Cy7 CD8
NC
1.75
I
6.05
CL
6.47
PE-Cy7 CD16

Supplementary Figure 1. Flow cytometry gating.

PBMCs were isolated and after 2-NBDG incubation, the cells were stained with a lymphoid and a myeloid panel of antibodies. A representative example of the gating performed with the FlowJo v10 software is shown here with the gating of $CD4^+$ T cells (CD4+), $CD8^+$ T cells (CD8+), NK cells (NK), classical (CL), non-classical (NC) and intermediate (I) monocytes.

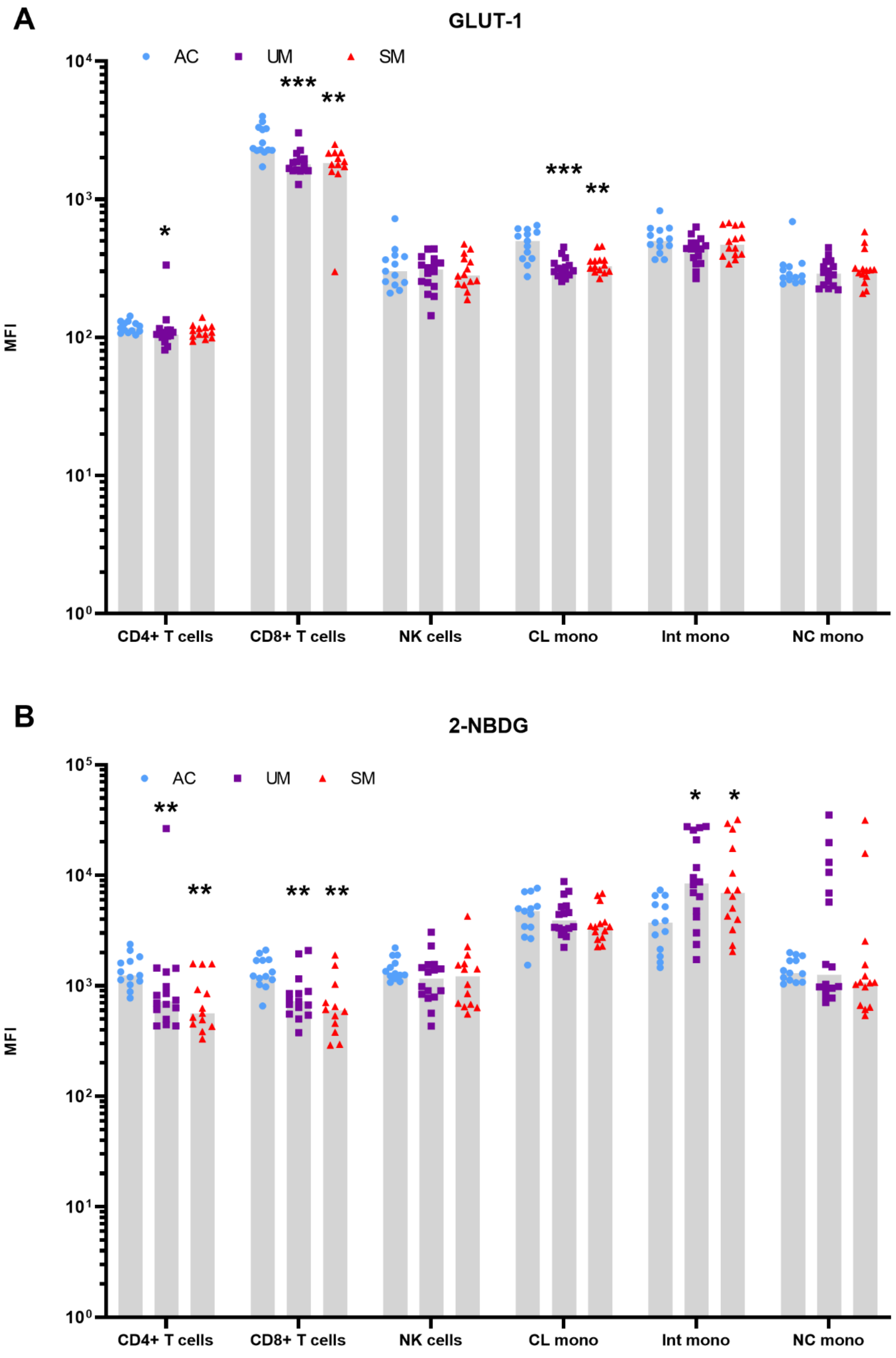
A
GLUT-1
AC
UM
SM
MFI
10^4
10^3
10^2
10^1
10^0
***
**
*
***
**
CD4+ T cells
CD8+ T cells
NK cells
CL mono
Int mono
NC mono
B
2-NBDG
AC
UM
SM
MFI
10^5
10^4
10^3
10^2
10^1
10^0
**
**
**
**
*
*
CD4+ T cells
CD8+ T cells
NK cells
CL mono
Int mono
NC mono

Supplementary figure 2. Expression of GLUT1 and uptake of 2-NBDG in different cell types. PBMCs were isolated, 2-NBDG uptake was allowed for 30 min and flow cytometry was performed. **(A)** Median fluorescence intensity (MFI) of GLUT-1 for the indicated subsets. **(B)** MFI of 2-NBDG for the indicated subsets. **(A-B)** Each symbol represents data from an individual. Bars represent group medians and analysis was by Mann-Whitney U-test. Asterisks above individual data sets indicate statistical differences compared to the HC group. * $p<0.05$, ** $p<0.01$, *** $p<0.001$. NK: Natural Killer; CL mono: classical monocyte; Int mono: Intermediate monocyte; NC mono: Non-classical monocyte. AC n=13; UM n=16; SM n=14. For $CD4^+$ T cells: UM n=15; SM n=13 and for $CD8^+$ T cells: UM n=15; SM n=11.

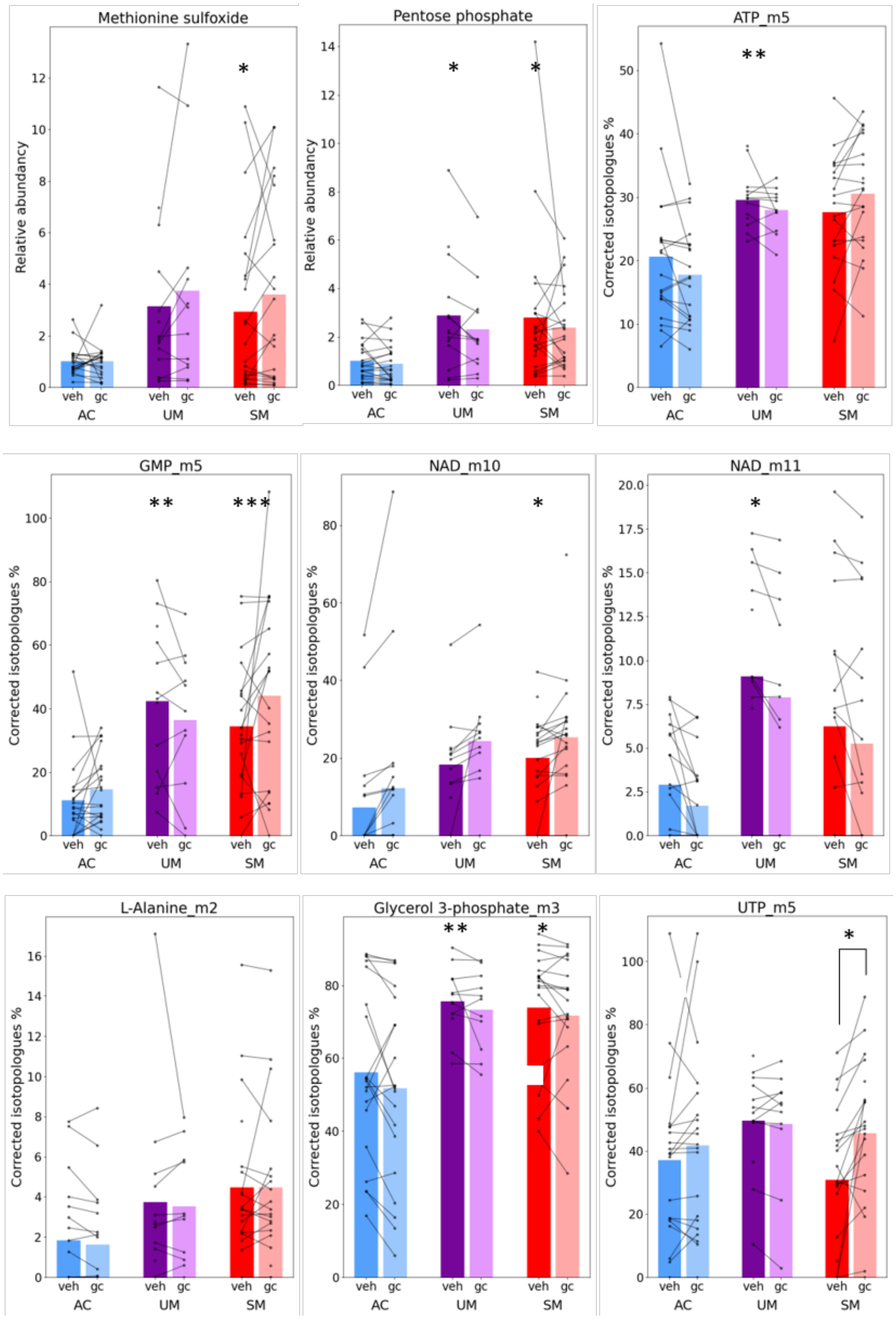

Supplementary figure 3: Relative abundancies and corrected isotopologue percentages of selected metabolites. PBMCs were isolated and incubated for 24 hours with [U-^{13}C]-glucose tracer and vehicle (veh) or 150 nM hydrocortisone (gc). Metabolic extracts were analyzed via mass spectrometry. Each symbol represents data from an individual. Bars represent the mean. Analysis by

t-test for comparison between the veh condition of different groups, indicated by asterisks above individual data sets. Analysis by paired t-test for GC-induced effects, indicated by asterisks above lines. Abundancies: AC n=20; UM n=14; SM n=22 veh. Isotopologues: AC n=20; UM n=13; SM n=20.
